# Ibuprofen‐Functionalized Alkyl *α*‐hydroxy Methacrylate‐Based Polymers

**DOI:** 10.1002/open.202500038

**Published:** 2025-06-04

**Authors:** Burcu Balaban, Seckin Altuncu, Aleyna Esenturk, Simay Denizkusu, Ece Sabuncu, Hande Sipahi, Duygu Avci

**Affiliations:** ^1^ Department of Chemistry Bogazici University Bebek 34342 Istanbul Turkey; ^2^ Department of Pharmaceutical Toxicology Faculty of Pharmacy Yeditepe University Istanbul 34775 Turkey

**Keywords:** alkyl *α*‐hydroxymethacrylates, drug design, ibuprofen, inflammation, polymer‐drug conjugates

## Abstract

Novel alkyl *α*‐hydroxymethacrylate‐based prodrugs are prepared to improve bioavailability, decrease toxicity, and control drug release. First, an ibuprofen (IBU)‐functionalized methacrylate (TBMA‐IBU) is synthesized from the reaction of *tert*‐butyl *α*‐bromomethacrylate with IBU. The free radical homopolymerization and copolymerization with poly(ethylene glycol) methyl ether methacrylate (*M*_n_ = 300), cleavage of *tert*‐butyl ester groups of the homopolymer and a copolymer with trifluoroacetic acid (TFA) gave new polymer prodrugs (*p*‐TBMA‐IBU, *p*‐TBMA‐IBU*‐co*‐PEGMA, *p*‐MA‐IBU, and *p*‐MA‐IBU*‐co*‐PEGMA), with IBU linked through ester bonds on the side chain. The release studies showed extended‐release of IBU (20–60% in 15 d), which can be triggered under the stimulation of lipase. The in vitro studies indicate that the representative polymers are effective in relieving inflammatory responses in RAW264.7 macrophage cells without any cytotoxicity. These results suggest that the synthesized polymers with controllable functionality can be promising IBU prodrugs.

## Graphical abstract

This work describes the synthesis and evaluation of novel alkyl *α*‐hydroxymethacrylate‐based ibuprofen‐functionalized prodrugs to improve the bioavailability, decrease toxicity, and control drug release.

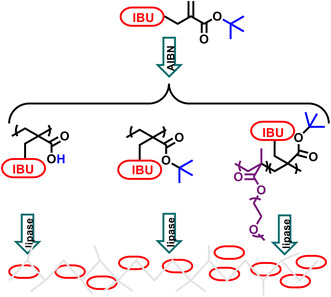

## 1 Introduction

Nonsteroidal anti‐inflammatory drugs (NSAIDs) are commonly used for the treatment of inflammation, pain, and arthritis.^[^ ^1^ ^]^ The anti‐inflammatory effects are associated with inhibition of cyclooxygenase (COX) enzymes, COX‐1 and COX‐2, which leads to a decrease in the production of prostaglandin, an important signaling molecule in the inflammation.^[^ ^2^ ^]^ Although nonselective NSAIDs inhibit both COX‐1 and COX‐2, selective NSAIDs preferentially inhibit COX‐2, which was expected to potentiate anti‐inflammatory effects and reduce adverse effects. Since there is a correlation between inflammation and the development of cancer, NSAIDs also have great potential in the prevention and treatment of several types of cancers.^[^ ^3^, ^4^, ^5^, ^6^ ^]^

Diclofenac and ibuprofen, followed by naproxen, indomethacin, piroxicam, and ketoprofen, are the most widely used NSAIDs.^[^ ^7^ ^]^ However, long‐term use of these drugs can cause a variety of side effects such as heart attack, stroke, and stomach ulcers, due to the lowered prostaglandin levels in the entire body. In order to eliminate side effects and improve other properties of NSAID administration, localized and targeted delivery are alternative methods of treatment.^[^ ^7^ ^]^ Physical incorporation of NSAIDs into polymeric micelles/nanoparticles was investigated,^[^ ^8^ ^]^ but sometimes this method showed low drug loadings (<30%) and burst release.^[^ ^9^ ^]^ A promising strategy is the “polymeric prodrug” approach by covalently attaching the anti‐inflammatory drugs onto the backbone of a polymer.^[^ ^10^, ^11^ ^]^ These “polymeric prodrugs” can improve the bioavailability of a drug, decrease their toxicity, facilitate administration, better control of drug release, or deliver the drug to specific tissues.^[^ ^2^ ^]^ “Polymeric drugs” can be synthesized by modification of natural (e.g., hydroxyethyl cellulose and dextran)^[^ ^12^, ^13^ ^]^ or synthetic polymers (e.g., polyethylene glycol and polyacrylic acid) with NSAIDs^[^ ^14^, ^15^, ^16^, ^17^ ^]^ or polymerization of monomeric drugs.^[^ ^9^, ^18^, ^19^, ^20^, ^21^, ^22^, ^23^, ^24^, ^25^, ^26^, ^27^, ^28^, ^29^, ^30^, ^31^ ^]^ Second method generally involves free radical polymerization of NSAID functional (meth)acrylates or (meth)acrylamides^[^ ^19^, ^20^, ^21^, ^22^, ^23^, ^24^, ^25^, ^26^ ^]^ or polycondensation reaction of drug molecules containing two functional groups.^[^ ^9^, ^28^, ^29^ ^]^ Among the last two, free radical polymerization significantly increases the drug loading, while polycondensation reaction cannot produce a high molecular weight polymer.

The development of safe anti‐inflammatory polymeric prodrugs targeting the inflammatory tissues and having the desired rate of release for control of drug administration remains challenging. It is known that inflammatory tissues have acidic pH (≈4.4–5.6), overexpressed levels of enzymes (e.g., cholesterol esterase (CE) and cyclooxygenases), and enhanced permeability (pathologic vessels in rheumatoid arthritis are similar to that in tumors) to macromolecules compared to normal tissues.^[^ ^10^, ^32^, ^33^, ^34^ ^]^ When inflammation occurs, a mass of macrophages accumulates around the inflammatory tissues and can secrete a large amount of CE,^[^ ^35^ ^]^ which has the ability to cleave polyester and poly(ether‐urethane)s bonds.^[^ ^36^ ^]^ In this case, by grafting the carboxylic acid moieties of NSAIDs onto a polymer chain through ester bonds, the release of NSAIDs can be controlled by cleaving the ester bonds via CE or lipase.^[^ ^32^, ^37^ ^]^ This unique characteristic in inflammatory tissues can be applied to manipulate the release of NSAIDs that are grafted onto prodrugs through ester linkages.

Another important issue of designing a polymeric prodrug is the rate of drug release, which depends on the stability of the drug‐polymer linkage and molecular weight, hydrophilicity, and biodegradability of the polymer.^[^ ^14^, ^15^, ^20^, ^24^, ^25^, ^27^, ^38^, ^39^ ^]^ Typical linkage groups are esters, anhydrides, or amides. For example, Leonard et al. showed that the rate of drug release can be controlled by tailoring the type of spacer and hydrolyzable linker to IBU and naproxen using polyethylene‐based polymeric prodrugs.^[^ ^27^ ^]^ Macromonomers with a biodegradable lactide spacer between IBU and methacrylate were emulsion polymerized to produce pH‐sensitive drinkable nanoparticles for oral delivery of IBU.^[^ ^40^ ^]^ PEG derivatives of ibuprofen^[^ ^14^ ^]^ and naproxen^[^ ^15^ ^]^ with different molecular weights were prepared by esterification reactions to prolong their anti‐inflammatory effects. Amphiphilic diblock copolymers of IBU micellar prodrugs were prepared by reversible addition fragmentation transfer (i) polymerization of an acrylamide derivative of IBU using PEG‐CTA as chain transfer agent^[^ ^23^ ^]^ or (ii) copolymerization of IBU‐based vinyl monomer and PEGMA^[^ ^18^ ^]^ and their anticancer properties were investigated with or without loading drugs such as doxorubicin and paclitaxel.^[^ ^18^ ^]^ Various methacrylate‐based monomeric prodrugs were prepared by functionalization of 2‐hydroxyethyl(meth)acrylate (HEMA or HEA), 2‐hydroxypropylmethacrylate (HPMA) or 2‐hydroxyethyl acrylamide with NSAIDs and converted to polymeric prodrugs by homo‐ and copolymerization with different vinyl monomers.^[^ ^26^, ^38^ ^]^ These polymeric drugs have two hydrolyzable ester bonds on each side of the spacer group, which can be cleaved into not only drugs but also hydroxyethyl or hydroxypropyl esters of drugs, depending on release conditions.^[^ ^21^, ^22^, ^24^, ^25^ ^]^ Davaran and Entezami prepared acryloyl‐ and methacryloylethyl ester and amide monomers of ibuprofen and indomethacin.^[^ ^20^ ^]^ It was shown that these prodrugs could be readily cleaved between the drug and spacer using either ester or amide linkages.

The aim of this work was to develop IBU functionalized alkyl *α*‐hydroxymethacrylate‐based prodrugs and evaluate the influence of their structures on the release properties. Alkyl *α*‐hydroxymethacrylates or alkyl *α*‐bromo(chloro)methacrylates allow incorporation of different functional groups via ester linkages through their reactions with carboxylic acids. The presence of other alkyl (methyl, ethyl, *tert*‐butyl) ester functionality in the side chain controls hydrophilicity, hence the solubility properties of these derivatives and also the properties of the polymers they get incorporated into. Therefore, there is no need to prepare copolymers with monomers with different hydrophilicity to manipulate this property, as seen in prodrugs of methacrylates in the literature. First, monomer (IBU)‐functionalized methacrylate (TBMA‐IBU was prepared by the esterification of carboxyl groups of IBU with *tert*‐butyl *α*‐bromomethacrylate (TBBr). Then, the prodrugs with ester‐linked IBU on their side chain were prepared by homopolymerization of TBMA‐IBU, copolymerization of TBMA‐IBU with PEGMA (*p*‐TBMA‐IBU*‐co*‐PEGMA), and cleavage of *tert*‐butyl ester groups of the homopolymer and one of the copolymers to give carboxylic acid functionality (*p*‐MA‐IBU, *p*‐MA‐IBU*‐co*‐PEGMA). The polymers (*p*‐TBMA‐IBU and *p*‐MA‐IBU) can be utilized to observe the effect of the side chain on release properties, the copolymers enable to control of drug loading. The inflammatory responses in RAW264.7 macrophage cells and cytotoxicities of the two polymeric prodrugs were investigated.

## 2 Results and Discussion

### 2.1 Synthesis and Characterization of the Monomeric and Polymeric Prodrugs

**Figure 1:**
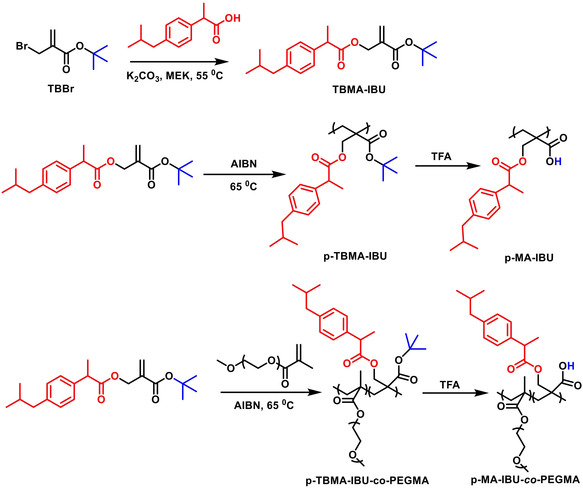
Synthesis of TBMA‐IBU and its polymer conjugates.

A methacrylate (TBMA‐IBU) containing an ester‐linked IBU unit was synthesized from the nucleophilic substitution reaction of TBBr^[^ ^41^, ^42^ ^]^ with IBU (**Figure** **1**). This monomer was obtained as a colorless liquid in 68% yield. **Table** **1** summarizes the solubility of TBMA‐IBU and IBU as reference, in various solvents. TBMA‐IBU exhibits similar solubility behavior to IBU, soluble in highly polar (methanol), slightly polar (acetone, dichloromethane (DCM), diethyl ether), and nonpolar (hexane) organic solvents; however, they both are insoluble in water.

**Table 1:** Solubility of IBU, TBMA‐IBU and its polymers.

Monomer/Polymer	H_2_O	MeOH	(C_2_H_5_)_2_O	DCM	THF	Hexane
IBU	–	+	+	+	+	+
TBMA‐IBU	–	+	+	+	+	+
*p*‐TBMA‐IBU	–	–	+	+	+	+
*p*‐MA‐IBU	–	–	+	–	+	–
TBMA‐IBU*‐co*‐PEGMA (50:50 mol%)	–	+/−	+	+	+	–
TBMA‐IBU*‐co*‐PEGMA (20:80 mol%)	–	+	+	+	+	–
MA‐IBU*‐co*‐PEGMA (20:80 mol%)	–	+/−	–	+	+	–

**Figure 2:**
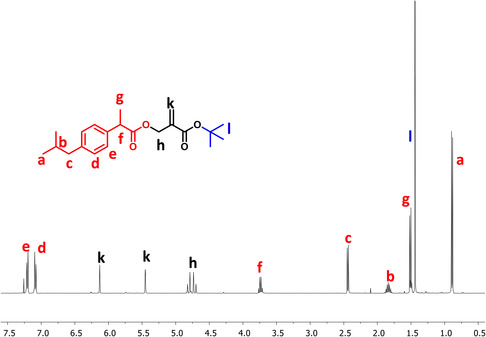
^1^H NMR spectrum of TBMA‐IBU.

**Figure 3:**
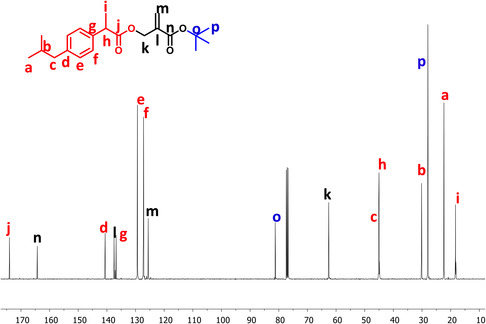
^13^C NMR spectrum of TBHMA‐IBU.

The purity of the synthesized monomer was confirmed by ^1^H and ^13^C NMR. The ^1^H NMR showed characteristic peaks for IBU, aromatic protons at 7.09 and 7.21 ppm, and isobutyl protons at 0.89 (CH_3_), 1.84 (CH), and 2.44 (CH_2_) ppm. In addition, the peaks at 1.44 (*tert*‐butyl), 4.76 (CH_2_), and 5.45, 6.13 (=CH_2_) ppm belong to the methacrylate structure (**Figure** **2**). In the ^13^C NMR spectrum of TBMA‐IBU, the peaks at 27.96 and 81.24 ppm are typical of *tert*‐butyl carbons. The peaks at 18.31, 22.35 (two different methyl carbons), 30.16, 45.12 (two different methine) ppm are assigned to the carbons of IBU functionality (**Figure** **3**). The Fourier‐transform infrared (FTIR) spectrum of TBMA‐IBU displays two different carbonyl stretches at 1720 and 1740 cm^−1^ (the first one conjugated with the double bond) and double bond stretch at 1640 cm^−1^ (Figure S1, Supporting Information).

**Figure 4:**
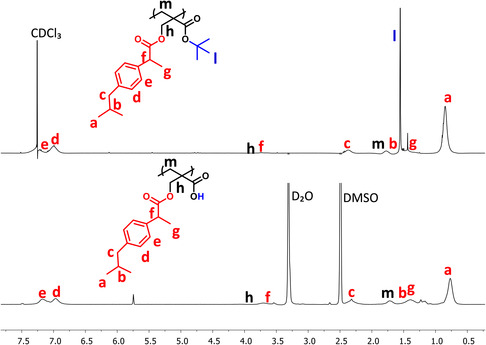
^1^H NMR spectra of *p*‐TBMA‐IBU (top) and *p*‐MA‐IBU (bottom).

Polymeric drug of TBMA‐IBU was synthesized by its free radical polymerization in bulk using AIBN as the initiator (Figure 1). The rate of polymerization of this monomer was quite fast; e.g., it reached 50% conversion (after purification) within 2 h. The results are shown in **Table** **2** where yield, molecular weights, molecular weight distributions (*M*_w_ */M*_n_), and glass transition temperatures (*T*_g_) are summarized. ^1^H NMR spectrum of the polymer showed disappearance of the characteristic peaks of double bonds in the monomer (**Figure** **4**). Also, the spectrum of the prodrug showed broadened peaks compared to TBMA‐IBU.

**Table 2:** Polymerization conditions and characteristics of the synthesized polymers.

M1	M2	Solvent	[M]	AIBN [wt%]	Time [min]	Temp. [°C]	Yield [%]	T_g_	M_n_	*Đ* a)
TBMA‐IBU	–	bulk	–	1	120	60	50	118	74 300	2.92
TBMA‐IBU	–	bulk	–	1	1440	60	73	–	–	–
TBMA‐IBU	–	chloroform	2.2	1.5	120	65	40	100	15 400	3.34
TBMA‐IBUb)	PEGMA	methanol	1.5	1.5	1110	60	XPc)	–	–	–
TBMA‐IBUb)	PEGMA	chloroform	2.2	1.5	270	65	XPc)	–	–	–
TBMA‐IBUb)	PEGMA	chloroform	1	1.5	120	65	36	58	206 100	1.25
TBMA‐IBUd)	PEGMA	chloroform	1	1.5	90	65	29	–	130 800	2.45
TBHMA‐IBUe)	PEGMA	chloroform	1	1.5	90	65	24	–	168 500	1.05

a) *Đ* = dispersity index

b) 50 mol%

c) crosslinked polymer

d) 80 mol%

e) 20 mol%.

**Figure 5:**
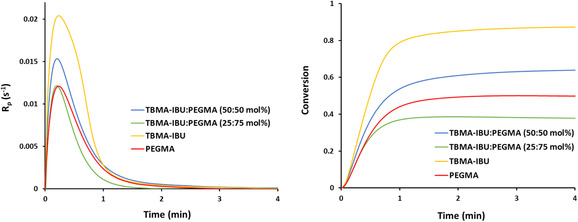
*R*_p_ versus time and conversion versus time plots of TBMA‐IBU, PEGMA, and TBMA‐IBU:PEGMA (25:75 and 50:50 mol%).

In the FTIR spectrum of the polymer, the alkene peak at 1643 cm^−1^ of the monomer is not present. Also, we observed only one type of carbonyl stretch at 1728 cm^−1^ due to the disappearance of conjugation after polymerization (Figure S2, Supporting Information). The number‐average molecular weight (*M*_n_), weight‐average molecular weight (*M*_w_), and polydispersity (*M*_w_ */M*_n_) were found to be 74.300, 217.000 g mol^−1^, and 2.92. These high molecular weights are consistent with the other ester derivatives of alkyl *α*‐hydroxymethacrylates. Our previous work showed that ester derivatives are very reactive and give high molecular weight polymers due to the Trommsdorff (autoacceleration) effect.^[^ ^43^ ^]^ This effect is due to a decrease in termination relative to propagation caused by an increase in viscosity of the polymerization medium. The polymer molecular weight affects their biodistribution and pharmacokinetic profile. The higher the molecular weight of polymers, the longer the blood circulation duration, which increases their accumulation in inflamed tissues. In order to study the effect of molecular weight on the release properties of the prodrug, we also conducted solution polymerization of TBMA‐IBU in chloroform using AIBN. As expected, *M*_n_ values of the polymers (15 400 g mol^−1^) were found to be lower than those found for bulk polymers. Furthermore, in order to evaluate the reactivity of TBMA‐IBU, it was also photopolymerized using 1 wt% 2,2’‐dimethoxy‐2‐phenylacetophenone (DMPA) at 30 °C (**Figure** **5**). The rate and conversion of polymerization were quite high; maximum conversions of 87% were reached within 3.28 min.

In the literature, IBU‐functionalized monomers were copolymerized with methacrylic acid to obtain pH sensitivity in the digestive system. The carboxylic acid groups enable solubilization of the polymer in the intestinal tract. Thus, drugs that produce side effects in the stomach could pass through the acidic gastric region without undergoing any change. In order to introduce carboxylic acid groups to our IBU‐delivery system, *tert*‐butyl ester groups of *p*‐TBMA‐IBU were cleaved by TFA, which is the most common deprotecting reagent in the literature. The conversion of the ester to the carboxylic acid was verified by ^1^H NMR spectrum, indicating almost complete disappearance of *tert*‐butyl units (Figure 4). In the FTIR spectrum of this polymer (*p*‐MA‐IBU), the main features are a broad C = O stretching between 1725 and 1680 cm^−1^ due to carboxylic acid dimer formation and a C‐*O*‐C bending at 1100 cm^−1^ (Figure S2, Supporting Information). Other signals are identical to those of the *p*‐TBMA‐IBU. In contrast to *p*‐TBMA‐IBU. which shows good solubility in nonpolar and low polarity solvents such as hexane and DCM, *p*‐MA‐IBU was insoluble in these solvents.

The copolymerization of TBHMA‐IBU with a biocompatible monomer, PEGMA, in 80:20, 50:50, and 20:80 mol ratios was also carried out in this work (Figure 1 and Table 2). The copolymer structures were confirmed by ^1^H NMR (Figure S3 and S4, Supporting Information); the dominant PEG signals appear at 3.64 ppm, the methoxy protons at 3.4 ppm, the methyl protons of PEGMA main‐chain at 1.0–1.2 ppm while the peaks around 7.0–7.4 ppm correspond to the aromatic protons of IBU (Figure S3, Supporting Information). The composition of the copolymers was determined by integration of aromatic peaks with respect to methoxy protons of PEGMA, and the mol ratio of PEGMA was calculated as 62–67% for 50:50 mol% copolymer and ≈85% for 20:80 mol% copolymer. We observed crosslinked polymer formation during two copolymerization reactions, which were carried out at high monomer concentrations (1.5 and 2.2 M) when the ratio of TBHMA‐IBU:PEGMA is 50:50 mol%. This result is due to PEGMA since homopolymerization of TBHMA‐IBU at high concentration (2.2 M) gave soluble polymers. In the literature, it is known that polyethylene oxide (PEO) is used as a hydrogen donor to Type II photoinitiators, and also thermal oxidation of PEO is possible, both reactions give free radicals.^[^ ^44^, ^45^ ^]^ Therefore, at high concentrations of PEGMA, we expect radical formation on PEG units, which leads to crosslinking during polymerization.

The cleavage of the *tert*‐butyl ester groups of one of the copolymers (TBMA‐IBU*‐co*‐PEGMA (20:80 mol%)) by TFA gave a carboxylic acid functionalized prodrug, MA‐IBU*‐co*‐PEGMA (20:80 mol%) (Figure S4, Supporting Information).

The FTIR spectrum of one of the copolymers, TBMA‐IBU*‐co*‐PEGMA (50:50 mol%) (Figure S2, Supporting Information) shows the ester carbonyl groups at 1730 cm^−1^. The absence of characteristic C = C stretching peak around 1630 cm^−1^ in the copolymer spectrum indicated successful copolymerization. The number‐average molecular weight (*M*_n_), weight‐average molecular weight (*M*_w_), and polydispersity (*M*_w_ */M*_n_) of this copolymer were found to be 206.100, 257.600 g mol^−1^, and 1.25. The copolymerization rate of TBHMA‐IBU with PEGMA was also investigated using photodifferential scanning calorimetric (DSC) (Figure 5). TBHMA‐IBU showed a faster polymerization rate and conversion (*R*_p_ = 0.02039 s^−1^, conversion = 87.5%) than that of PEGMA (*R*_p_ = 0.01208 s^−1^, conversion = 50.0%). Its maximum rate of polymerization and conversion was found to decrease with an increase in concentration of PEGMA.

**Figure 6:**
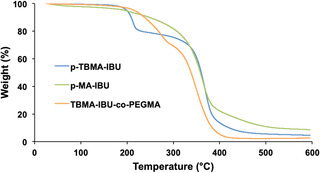
Thermal degradation of *p*‐TBMA‐IBU, *p*‐MA‐IBU, and *p*‐TBMA‐IBU*‐co*‐PEGMA (50:50 mol%).

The glass transition behavior of the polymers studied by DSC analysis. *p*‐TBMA‐IBU showed a glass transition temperature of 118 °C, however, we could not observe any apparent *T*_g_ for other polymers. The thermal stability of the polymeric drugs was investigated by thermogravimetric analysis (TGA) under nitrogen atmosphere (**Figure** **6**). *p*‐TBMA‐IBU showed two distinct stages of thermal degradation at temperatures of ≈200 and 320 °C. The first stage of degradation (19%) was attributed to the cleavage of the *tert*‐butyl groups,^[^ ^46^ ^]^ and the second stage was ascribed to the main chain degradation. TBMA‐IBU*‐co*‐PEGMA (50:50 mol%) also showed approximately similar behavior to *p*‐TBMA‐IBU, two‐step degradation at 215 and 305 °C (*p*‐PEGMA shows one‐step weight loss at about 300 °C^[^ ^47^ ^]^). However, degradation of *p*‐MA‐IBU occurred at one stage starting from 200 °C, which also confirms the absence of *tert*‐butyl groups on this polymer. The char yields also differ for *p*‐MA‐IBU (9%) and *p*‐TBMA‐IBU (5%). The higher char yield of *p*‐MA‐IBU can be explained by crosslinking due to anhydride formation.

The amphiphilic polymers can self‐assemble to form nanoparticles in aqueous solution. It was expected that copolymers (TBMA‐IBU*‐co*‐PEGMA and MA‐IBU*‐co*‐PEGMA) may form nanoparticles with the hydrophobic segments (Ibuprofen) forming a core and hydrophilic segments (PEG and COOH) a shell. In order to investigate the formation of nanoparticles from MA‐IBU*‐co*‐PEGMA (20:80 mol%), the nanoprecipitation method (dropwise addition of water to THF solution of the copolymer) was used. The morphology of the nanoparticles was observed with SEM (Figure S5, Supporting Information). The SEM pictures of the nanoparticles showed spherical morphology. The observed particle sizes were 25–64 nm.

### 2.2 Ibuprofen Release from the Prodrugs

There are different factors affecting the rate of hydrolysis of drug pendant polymers, such as the structure of polymers, chemical bonds between polymer and drug, spacer length between polymer and drug, and its hydrophilicity, molecular weight, and hydrophilicity of polymers, steric effect of neighboring groups, and also hydrolysis conditions. In all our polymers, IBU is attached to the polymer via ester linkages. However, polymers have different functional groups at their side chain, which affect hydrophilicity, pH dependence, and steric hindrance. Also, molecular weights of the polymers range from 15 000 to 200 000.

In order to observe the performance of our prodrugs under physiological conditions, the drug release studies were performed in phosphate‐buffered saline (PBS) (pH 7.4) media with or without esterase (lipase from Pseudomonas cepacia) at 37 °C under shaking. The lipase was used to simulate enzyme‐rich body fluid during inflammation.^[^ ^32^ ^]^ The concentration of the enzyme was selected as 10 U mL^−1^, which is comparable to the esterase level in human serum.^[^ ^48^ ^]^ Since the polymers were not soluble in water, the hydrolysis was performed in a heterogeneous system using dialysis tubing (MWCO = 1000), which is permeable to low molecular weight IBU. The amount of the released IBU passing to the external solution at different time intervals was determined from its absorbance at 264 nm using a UV spectrophotometer and calculated from the calibration curve.

**Figure 7:**
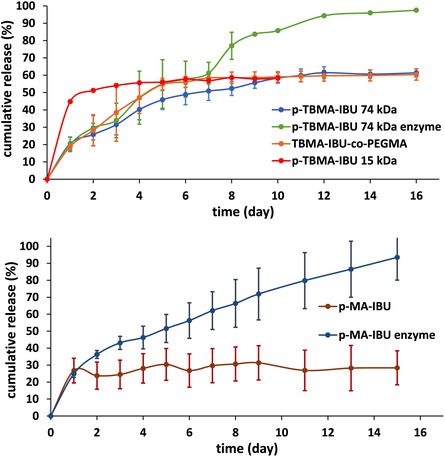
The release profiles of IBU from *p*‐TBMA‐IBU, *p*‐TBMA*‐co*‐PEGMA (50:50 mol% in feed) (top), and *p*‐MA‐IBU with or without lipase (bottom).

**Figure** **7** shows the cumulative IBU release from the prodrugs as a function of time. *p*‐TBHMA‐IBU has two hydrolyzable ester linkages, between the *tert*‐butyl group and the polymer backbone, and the drug and polymer backbone. The *tert*‐butyl ester group linkage is expected to be less susceptible to hydrolysis due to the steric effect. As shown in Figure 7, no significant burst release of IBU was observed from *p*‐TBMA‐IBU (*M*_n_ = ≈74 000), which is the drawback observed for small‐molecule drugs. This prodrug showed very slow release of IBU in PBS, reaching 46% in 5 d, then the release rate decelerated (62% on day 12), and the total amount of released IBU stayed almost constant after the 12th day. It is worth noticing that the release of pure drug IBU was 77% in 1 h. A more hydrophilic prodrug, *p*‐TBMA‐IBU*‐co*‐PEGMA (50:50 mol% in feed) (*M*_n_ = ≈200 000) containing 37 w w^−1^% IBU showed a faster initial release rate, reaching 55% in 5 d which can be attributed to the fact that hydrolysis of accessible ester bonds is easy because hydrophilicity of PEGMA facilitates water entrance. However, the total amount of released IBU was similar to *p*‐TBMA‐IBU, which can be explained by almost 3 times higher molecular weight of this polymer, causing steric hindrance. The lower molecular weight (*M*_n_ = ≈15 000) *p*‐TBMA‐IBU was hydrolyzed more rapidly, with an IBU release rate of almost twofold higher than *p*‐TBMA‐IBU (*M*_n_ = ≈74 000) and *p*‐TBMA‐IBU*‐co*‐PEGMA (*M*_n_ = ≈200 000), reaching ≈60% in 2 d. The copolymer *p*‐TBMA‐IBU*‐co*‐PEGMA (20:80 mol% in feed) (*M*_n_ = ≈168 000) showed 10% higher final release at PBS in 13 d compared to *p*‐TBMA‐IBU*‐co*‐PEGMA (80:20 mol% in feed) (*M*_n_ = ≈130 000), indicating the importance of hydrophilicity.

In comparison, the lipase presence showed a difference in released amount in 4 d and IBU release accelerated especially after 8 d. After 16 d, the cumulative release from *p*‐TBMA‐IBU derivatives was around 60% at PBS, whereas 97% in the presence of lipase. As seen from Figure 7b, ≈26% of IBU was released from *p*‐MA‐IBU in 1 day, and it stayed almost constant for 15 d at PBS. Although the first day release was similar, a remarkable increase and control were achieved in enzyme‐catalyzed degradation. The copolymer *p*‐MA‐IBU*‐co*‐PEGMA (20:80 mol% in feed) (*M*_n_ = ≈168 000) showed 64% release at PBS in 16 d.

### 2.3 In Vitro Cytotoxicity

Nontoxic concentrations of TBMA‐IBU*‐co*‐PEGMA (20:80 mol% in feed) and MA‐IBU*‐co*‐PEGMA (20:80 mol% in feed) were determined by cell viability of 70% and above using the 3‐(4,5‐dimethylthiazol‐2‐yl)‐2,5‐diphenyltetrazolium bromide (MTT) assay. Both ibuprofen–functionalized polymers had no cytotoxic effects at concentrations up to 200 μM at 24 h as compared with control cells that received no treatment (**Table** **3**).

**Table 3:** Effects of ibuprofen‐functionalized polymers on the viability of RAW264.7 macrophage cells and on nitrite levels and % nitrite inhibition in RAW 264.7 cells stimulated with 1 μg mL^−1^ LPS.

Groups	Dose [μM]	Cell Viability [%]	Nitrite Level [μM]	Nitrite Inhibition [%]
Ctrl	–	108.78 ± 0.28	1.49 ± 0.26	–
LPS	–	100.00 ± 0.16	62.11 ± 0.59	–
L‐Name	100	93.53 ± 0.59	31.41 ± 1.07a)	49.42
Indomethacin	100	93.25 ± 0.55	29.82 ± 0.39a)	51.97
Ibuprofen	50	96.50 ± 0.65	46.07 ± 0.91a)	16.17
	100	93.36 ± 0.76	44.67 ± 0.70a)	28.07
	200	90.76 ± 0.68	41.83 ± 0.66a)	32.64
TBMA‐IBU*‐co*‐PEGMA (20:80)	50	90.20 ± 1.22	43.51 ± 0.65a)	29.95
	100	86.40 ± 1.41	38.78 ± 1.04a)	37.57
	200	82.37 ± 1.97	33.54 ± 0.30a)	45.99
MA‐IBU*‐co*‐PEGMA (20:80)	50	93.79 ± 1.73	44.26 ± 0.25a)	28.73
	100	88.19 ± 1.21	40.61 ± 1.92a)	34.63
	200	81.76 ± 0.82	37.72 ± 0.93a)	39.28

a) Ctrl: Control group treated with DMEM; LPS: Control group only stimulated with LPS; LPS: Lipopolysaccharides from E. coli; IND: Indomethacin (100 μM). Statistically significant differences were indicated for each compound versus LPS p < 0.001.

### 2.4 Anti‐Inflammatory Effect

**Figure 8:**
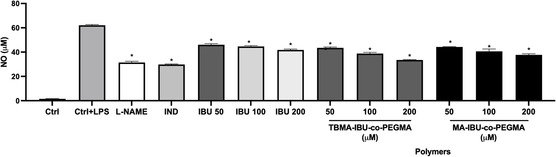
The effects of ibuprofen functionalized polymers at various doses (50, 100, and 200 μM) on nitrite production in LPS‐stimulated RAW 264.7 cells. Ctrl: Control group treated with DMEM; LPS: Control group only stimulated with LPS; LPS: Lipopolysaccharides from E. coli; Indomethacin: Indomethacin (100 μM); IBU: Ibuprofen. Statistically significant differences were indicated for each compound versus LPS (*p < 0.001.).

The ability of ibuprofen‐functionalized polymers to inhibit LPS‐induced nitrite production in RAW 264.7 cells was evaluated and compared with those of L‐name (N(G)‐Nitro‐L‐arginine methyl ester) (100 μM), a direct inhibitory agent of nitrite oxide, and indomethacin, a potent anti‐inflammatory agent, and ibuprofen (50–200 μM) serving as reference compounds. Treatment with LPS (1 μg mL^−1^) led to a significant increase in nitrite levels in the cell culture supernatant (Table 3). NO production after LPS stimulation increased to 62.11 ± 0.69 μM in the untreated cells. However, the polymers exhibited a concentration‐dependent reduction in LPS‐induced nitrite production, as shown in Table 3, **Figure** **8**. The treatment of L‐name and indomethacin significantly inhibited nitric oxide production by 49% and 51%, respectively (p < 0.001). In comparison of anti‐inflammatory activity of L‐name and indomethacin, ibuprofen showed less anti‐inflammatory effect. The polymers exert equal or better anti‐inflammatory effects in comparison to ibuprofen. Moreover, nitrite inhibition of TBMA‐IBU*‐co*‐PEGMA and MA‐IBU*‐co*‐PEGMA at their highest studied concentrations was ≈45% and 39%, respectively. Nitrite inhibition involves converting NO into L‐arginine via nitric oxide synthase (NOS), which is inhibited through competitive or noncompetitive mechanisms involving hydrogen bonds, van der Waals forces, electrostatic, and hydrophobic interactions.^[^ ^49^ ^]^ Hydrophobic interactions are crucial for the binding affinity and specificity of NOS inhibitors, enhancing their selectivity and effectiveness.^[^ ^50^ ^]^ In the literature, it was observed that the hydrophobic *tert*‐butyl groups promote interactions with cellular components or lipid membranes, enhancing the polymer's ability to modulate inflammatory pathways effectively.^[^ ^51^ ^]^ Intact *tert*‐butyl groups facilitate effective binding or stabilization within cells, thereby inhibiting nitrite production.^[^ ^52^ ^]^ The higher value of the first polymer may be due to bulky and hydrophobic *tert*‐butyl groups, enhancing interactions with cellular membranes and targets involved in NO production. In MA polymer, *tert*‐butyl groups are converted to methacrylic groups, introducing carboxylic acid functionalities that increase hydrophilicity and solubility. However, this may reduce interactions with hydrophobic targets or membranes compared to TBMA‐containing polymers.

## 3 Conclusions

In this work, we used a facile synthesis method based on alkyl *α*‐hydroxymethacrylates for the preparation of a highly reactive IBU‐functionalized monomeric prodrug. From this monomeric prodrug, we developed new polymeric IBU‐carrier systems with controllable functionality. Three prodrugs with an increased number of IBU units per polymer molecule with ester, carboxylic acid, and PEG side chains were synthesized in high yields. They have advantages over polymeric systems obtained by functionalization of polymers with drugs or encapsulation of drugs by micelles/nanoparticles both of which enable only limited loadings. The amount of cumulatively released IBU from the prodrugs was found to be low, 20–60% in 15 d, but increases to 100% in the presence of lipase, indicating enzyme‐responsive nature of these prodrugs. The two tested polymeric prodrugs (TBHMA‐IBU*‐co*‐PEGMA and MA‐IBU*‐co*‐PEGMA) did not affect the cell viability of RAW264.7 macrophages significantly at the concentrations of 50–200 μM, similar to ibuprofen. These prodrugs showed higher anti‐inflammatory activity compared to ibuprofen in a dose‐dependent manner. Therefore, we can say that the polymeric prodrugs based on alkyl *α*‐hydroxymethacrylates with anti‐inflammatory potential may increase bioavailability and decrease gastric side effects.

## 4 Experimental Section

4.1

4.1.1

##### Materials

*Tert*‐butyl *α*‐bromomethacrylate (TBBr) was synthesized according to literature procedures.^[^ ^41^, ^42^ ^]^ *Tert*‐butyl acrylate, paraformaldehyde, 1,4‐diazobicyclo[2.2.2]octane (DABCO), phosphorus tribromide (PBr_3_), 2,2’‐azobis(isobutyronitrile) (AIBN), 2,2’‐dimethoxy‐2‐phenylacetophenone (DMPA), hydroxyapatite, poly(ethylene glycol) methyl ether methacrylate (PEGMA, *M*_n_ = 300), TFA, 2‐hydroxyethyl methacrylate (HEMA), poly(ethylene glycol) diacrylate (PEGDA, *M*_n_ = 575 D), indomethacin, MTT, lipopolysaccharide (LPS) from *Eschrechia coli* 0111: B4 and the other reagents and solvents were obtained from Sigma Aldrich (St. Louis, Missouri, USA) and used as received without purification. Ibuprofen was a kind gift from Abdi İbrahim İlaç Sanayi & Ticaret A.Ş. Sodium nitrite was purchased from Fluka Chemika‐BioChemika (Buchs, Switzerland). RAW 264.7 mouse macrophages (ATCC TIB‐71 TM) were obtained from ATCC (Manassas, VA, USA). Dulbecco's modified Eagle's medium (DMEM) supplemented with 10% fetal bovine serum (FBS) and 1% penicillin (10.000 units mL^−1^) and streptomycin (10.000 mg mL^−1^) was purchased from Gibco, Thermo Fisher Scientific (Waltham, MA, USA).

##### Characterization Methods

Proton magnetic resonance (NMR) spectra were obtained using a Varian Gemini 400 MHz spectrometer in deuterated chloroform (CDCl_3_) or deuterated dimethyl sulfoxide (DMSO‐*d*_*6*_). FTIR spectra were collected by using Thermo Scientific Nicolet 380 spectrometer in the range of 4000–400 cm^−1^. Gel permeation chromatography measurements were carried out by a Malvern‐OmniSEC instrument using tetrahydrofuran (THF) as a solvent and polystyrene standard at METU Central Laboratory, Ankara, Turkey. DSC measurements were performed on a thermal analysis (TA) Instruments Q250 with a heating rate of 10 °C min^−1^. TGA was carried out on a TA Instruments Q500 under a nitrogen atmosphere with a heating rate 10 °C min^−1^. The morphologies of the polymer samples were examined using scanning electron microscopy (SEM) (FEI‐Philips XL30) under an accelerating voltage of 7.0 kV after sputter coating with a platinum layer.

##### Synthesis of Monomeric Prodrug (TBMA‐IBU)

TBBr (0.50 g, 2.3 mmol), IBU (0.51 g, 2.5 mmol), and K_2_CO_3_ (0.34 g, 2.5 mmol) were stirred in methyl ethyl ketone (6.2 mL) at 55 °C under nitrogen for 22 h. After filtration of the mixture, the solvent was removed under reduced pressure. The residue was diluted with dichloromethane (DCM) and extracted with water (3 × 5 mL). The organic phase was dried over anhydrous Na_2_SO_4_. After evaporation of the solvent under reduced pressure, TBMA‐IBU was obtained as a colorless liquid in 68% yield.

^1^H NMR (400 MHz, CDCl_3_, *δ*): 0.89 (d, J = 6.6 Hz, 6 H, C*H*_3_‐CH), 1.44 (s, 9 H, C*H*_3_‐C), 1.51 (d, J = 7.2 Hz, 3 H, C*H*_3_‐CH), 1.84 (dt, J = 13.5, 6.8 Hz, 1 H, (CH_3_)_2_‐C*H*‐CH_2_), 2.44 (d, J = 7.2 Hz, 2 H, C*H*_*2*_‐Ar), 3.74 (q, J = 7.2 Hz, 1 H, C*H*‐C = O), 4.76 (q, J = 8 Hz, 2 H, C‐C*H*_*2*_‐O), 5.45 (s, 1 H, C*H*_*1*_H_2_ = C), 6.13 (s, 1 H, CH_1_ *H*_*2*_ = C), 7.09 (d, J = 8 Hz, 2 H, Ar‐CH), 7.21 (d, J = 8 Hz 2 H, Ar‐CH) ppm.

^13^C NMR (100 MHz, CDCl_3_, *δ*): 18.31 (*C*H_3_‐CH), 22.35 (*C*H_3_‐CH), 27.96 (*C*H_3_‐C), 30.16 (*C*H‐CH_2_), 40.51 (CH_3_‐*C*H), 45.12 (CH‐*C*H_2_), 62.58 (*C*H_2_‐O), 81.24 (CH_3_‐*C*), 125.57 (*C*H_2_ = C), 127.21 (Ar‐*C*H), 129.33 (Ar‐*C*H), 136.77 (Ar‐*C*), 137.50 (CH_2_ = *C*), 140.73 (Ar‐*C*), 164.20 (C = O), 174.01 (C = O) ppm.

FTIR (attenuated total reflectance): 1740, 1720 (C = O), 1640 (C = C) cm^−1^.

##### Synthesis of Polymeric Prodrugs (*p*‐TBMA‐IBU, *p*‐MA‐IBU, *p*‐TBMA‐IBU‐*co*‐PEGMA, and *p*‐MA‐IBU‐*co*‐PEGMA)

Homo‐ and copolymerizations were carried out using standart freeze‐pump‐thaw method. The homopolymerization of TBMA‐IBU was carried out in bulk at 60 °C with AIBN (1 wt%) and in chloroform at 65 °C with AIBN (1.5 wt%) to give *p*‐TBHMA‐IBU. The polymers were purified by precipitation from CH_2_Cl_2_ into hot methanol and drying under vacuum. The copolymerization of TBMA‐IBU with PEGMA in 80:20, 50:50, and 20:80 mol ratio was conducted in chloroform at 65 °C with AIBN (Table 2). The copolymers were purified by precipitation into petroleum ether, followed by dialysis to remove unreacted TBMA‐IBU and PEGMA, and were obtained as colorless oil.

Excess TFA (0.22 mL, 2.9 mmol) was added dropwise to *p*‐TBMA‐IBU (0.137 g, 0.4 mmol) in an ice bath under nitrogen. The reaction took place at room temperature under nitrogen for 24 h. After removal of excess TFA, *p*‐MA‐IBU was obtained as a brownish‐pink solid. *p*‐MA‐IBU*‐co*‐PEGMA was obtained from *p*‐TBHMA‐IBU*‐co*‐PEGMA (20:80 mol% in feed) using the same procedure.

##### Photopolymerization Procedure

Photopolymerizations of TBMA‐IBU, PEGMA, and their mixtures (TBHMA:PEGMA, 50:50 and 25:75 mol%) were conducted by differential scanning calorimeter (TA Instruments DSC 250) using an Omnicure 2000 mercury lamp light source with a 320–500 nm filter. The monomer (3–4 mg) containing 1 wt% DMPA was exposed to irradiation at 30 °C under nitrogen for 5 min. The heat flow was monitored as a function of time, and the polymerization rate was calculated using Equation (1)

(1) $$\text{Rate}\;=\;\frac{\left(Q/s\right)M}{n\left(\Delta H_{p}\right)m}$$

where *Q/s* is the heat flow per second, *M* is the molar mass of the monomer, *n* is the number of double bonds per monomer molecule, Δ*H*_p_ is the heat evolved in the reaction and *m* is the mass of monomer in the sample. The theoretical heat for the total conversion of a methacrylate double bond is 55 kJ mol^−1^.

##### In Vitro Drug Release Studies

The drug release studies of the prodrug polymers were done in PBS in the presence or absence of the enzyme. Briefly, the pre‐weighed dry polymer sample (25–30 mg) and 3 mL PBS were transferred to dialysis membrane (1000 Da) and it was placed into a flask containing 200 mL of PBS with or without lipase (0.26 mg mL^−1^; 10 U/mL, from Pseudomonas cepacia) maintained at 37 °C and stirred at 150 rpm. At predetermined time intervals 3 mL of external solution was removed and an equal volume of fresh solution was added to the system. Each sample was analyzed by a Lasany UV–Vis double beam spectrophotometer LI‐2802 and the amount of released IBU was calculated using the absorbance at 264 nm according to the calibration curve. The linear correlation (R^2^ = 0.999) between the absorption and concentration of IBU was determined using known concentration samples (0.02, 0.05, 0.1, 0.3, 0.4 mg mL^−1^ of IBU in PBS).

##### Cell Viability Assay

To assess the impact of ibuprofen‐functionalized polymers on cell viability, the MTT assay was used. The RAW 264.7 mouse macrophages were cultured in complete medium (DMEM supplemented with 10% FBS, 1% penicillin (10 000 units mL^−1^), and streptomycin (10 000 mg/mL)), maintained at 37 °C with 5% CO_2_ in a humidified atmosphere.^[^ ^53^ ^]^ The cells were subcultured when they reached 80–90% confluence. RAW264.7 cells were initially seeded in 96‐well plates at a density of 5 × 10^4^ cells per well and incubated for 24 h in complete medium. Subsequently, the cells were treated with varying concentrations of the polymers (ranging from 12.5–100 μM) for another 24 h. The MTT reagent (0.5 mg/mL in PBS) was added to the cells and incubated for 2 h at 37 °C with 5% CO_2_. After removal of the culture supernatants, isopropanol (100 μL) was added to each well, and the absorbance at 570 nm was measured using a microplate reader (Thermo Fisher ScientificTM, Inc., Waltham, MA, USA). The cell viability assay was conducted three times, with each assay performed in triplicate (*n* = 9 in three separate experiments). A cell viability of cultures treated with polymers of less than 70% compared to untreated control cultures (medium group) is considered cytotoxic.^[^ ^54^ ^]^ The percentage of cell viability was calculated using Equation (2).

(2) $$\text{Cell viability }\left(\%\right)\text{ =100 }\times {\text{ OD}}_{\text{570e}}{\text{/OD}}_{\text{570b}}$$

where OD_570e_ is the mean value of the measured optical density of the 100% extract, and OD_570b_ is the mean value of the measured optical density of the blanks.

##### Anti‐Inflammatory Activity

The inhibition of NO production was assessed by measuring nitric oxide (NO) levels in the cell culture medium using the Griess reagent (0.1% N‐(1‐Naphthyl)‐ethylenediamine dihydrochloride in 5% phosphoric acid and 1% sulfanilamide).^[^ ^55^ ^]^ RAW 264.7 cells were seeded in a 96‐well plate at a density of 5 × 10^4^ cells per well and incubated for 24 h at 37 °C with 5% CO_2_. After pre‐treatment of the cells with various concentrations of ibuprofen‐functionalized polymers (12.5‐25‐50‐100 μM) for 2 h, and subsequent stimulation with 1 μg/mL of LPS (Lipopolysaccharide from Escherichia coli 0111: B4, Sigma, USA) for an additional 22 h, the cell culture supernatant was collected for analysis. The supernatant was combined with an equal volume of Griess reagent in a 96‐well plate and incubated for 10 min at room temperature in the dark. The color development corresponding to NO levels was measured at 540 nm using a microplate reader. The nitrite concentrations were determined using a sodium nitrite standard curve. Indomethacin (100 mM) was used as a positive control.

##### Statistical Analysis

Each experiment was carried out in triplicate. GraphPad Prism 9 was used for the statistical analyzes (GraphPad Software, Inc., San Diego, CA; The 8.4.3 version). Following the Tukey post hoc tests, one‐way ANOVA was used to determine group differences.

## Conflict of Interest

The authors declare no conflict of interest.

## Author Contributions

**Burcu Balaban**: investigation (equal); **Seckin Altuncu**: investigation (equal). **Aleyna Esenturk**: investigation (equal). **Simay Denizkusu**: investigation (equal). **Ece Sabuncu**: Investigation (equal). **Hande Sipahi**: investigation (equal); resources (equal). **Duygu Avci**: conceptualization (lead); Project administration (lead); Resources (lead); Supervision (equal); writing—review & editing (lead). **Burcu Balaban** and **Seckin Altuncu** contributed equally to this work.

## Supporting information

Supplementary Material

## Data Availability

The data that support the findings of this study are available from the corresponding author upon reasonable request.
