# Supplementary material for: Ibuprofen‐Functionalized Alkyl α‐hydroxy Methacrylate‐Based Polymers
Source: ChemistryOpen. 2025 Jun 4;14(9):e202500038. doi: 10.1002/open.202500038 (PMC12409833; doi:10.1002/open.202500038)
Supplement: Supplementary file 1 — Supplementary Material [file OPEN-14-e202500038-s001.pdf]

Supporting Information

Ibuprofen-functionalized alkyl α-hydroxy methacrylate-based polymers

Burcu Balaban[‡ a], *Seckin Altuncu*[‡ a], *Aleyna Esenturk*[a], *Simay Denizkusu*[a], *Ece Sabuncu*[b],

Hande Sipahi[b], *Duygu Avci*[* a]

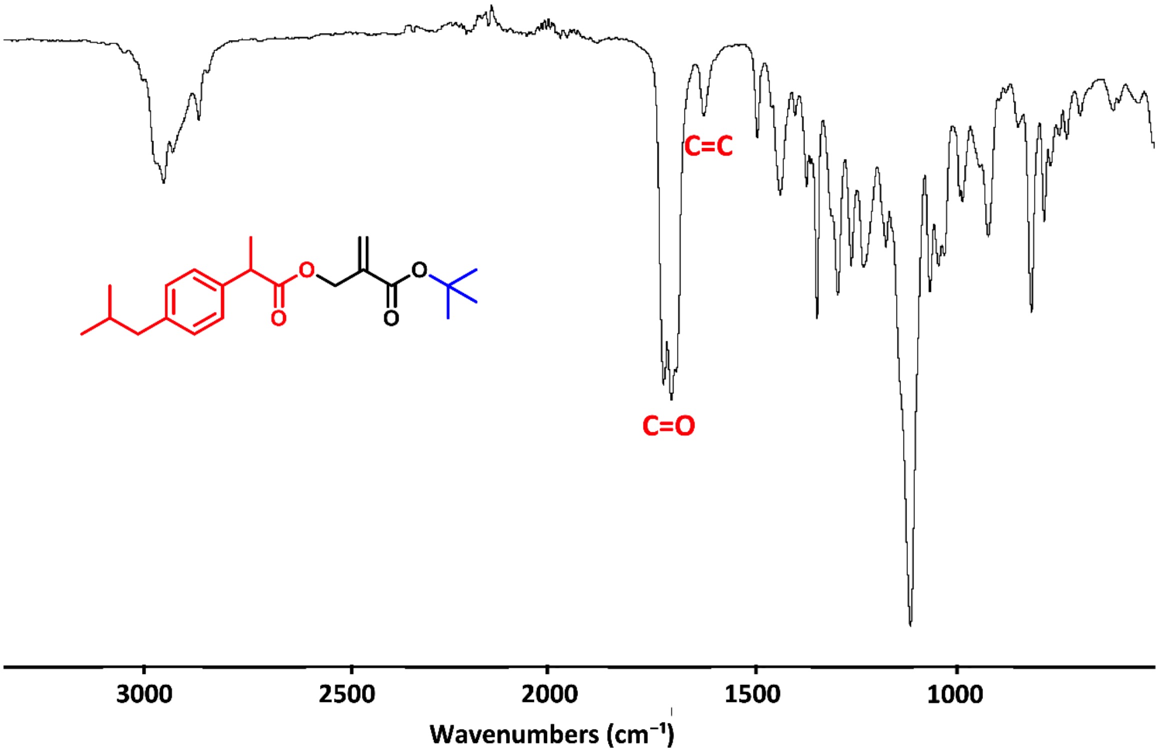

Figure S1. FT-IR spectrum of TBHMA-IBU

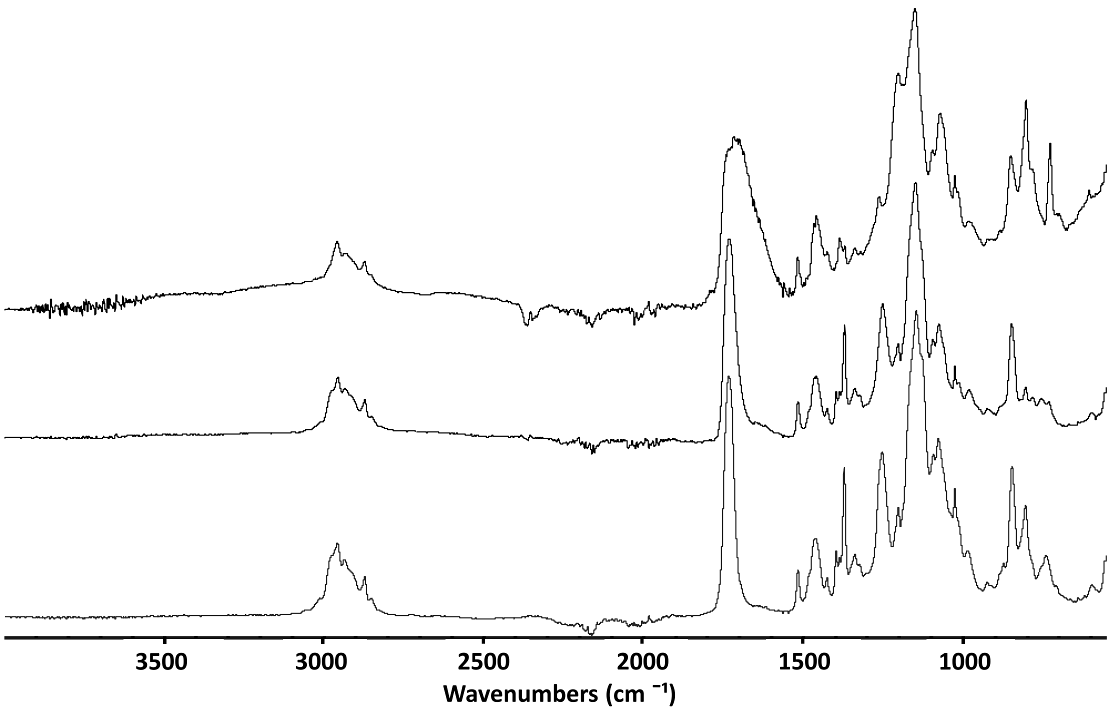

Figure S2. FT-IR spectra of p-TBMA-IBU, TBMA-IBU-*co*-PEGMA (50:50 mol% in feed), p-MA-IBU (from bottom to top).

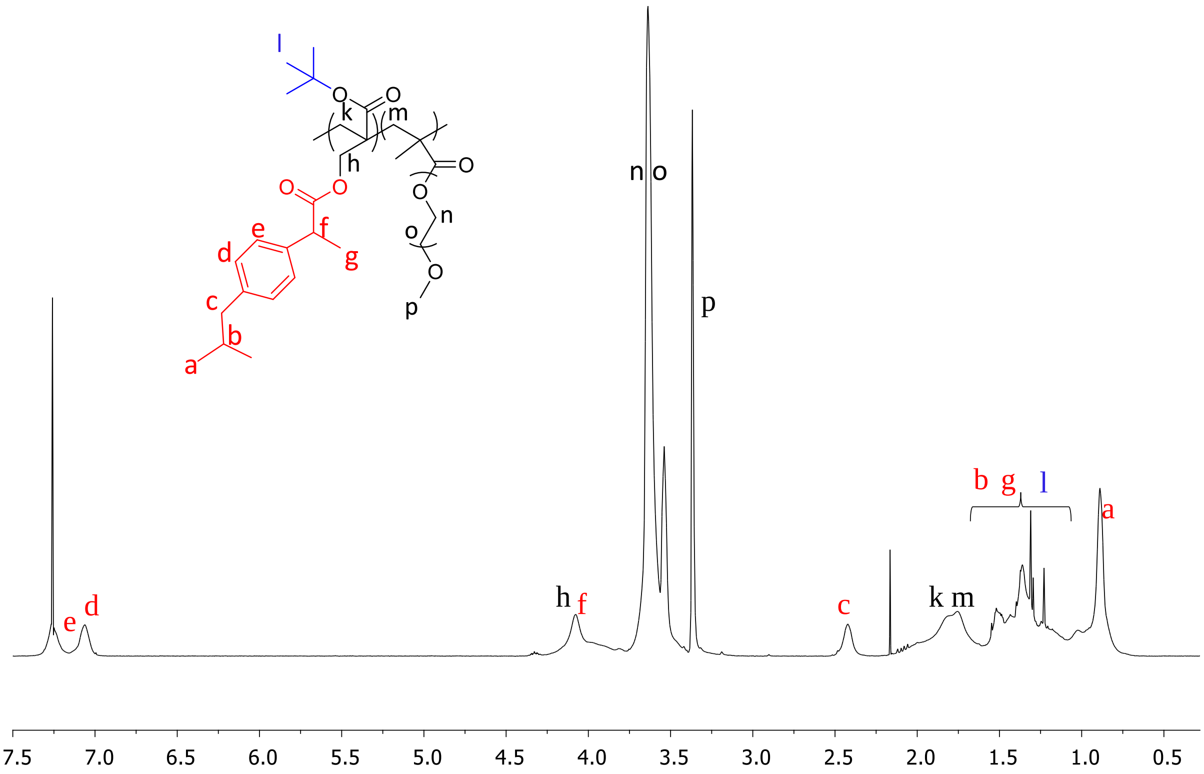

Figure S3. ^{1}H NMR spectrum of TBMA-IBU-*co*-PEGMA (50:50 mol% in feed)

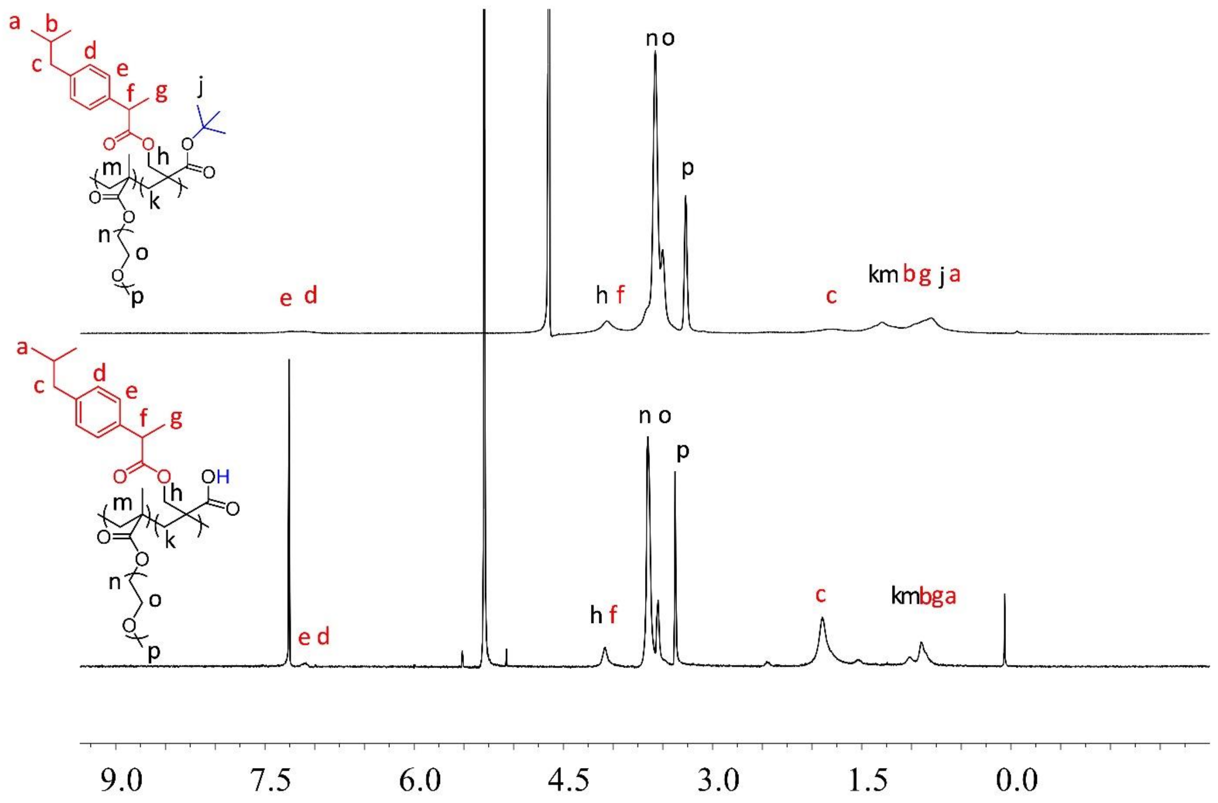

Figure S4. ^{1}H NMR spectra of TBMA-IBU-*co*-PEGMA and MA-IBU-*co*-PEGMA (20:80 mol% in feed)

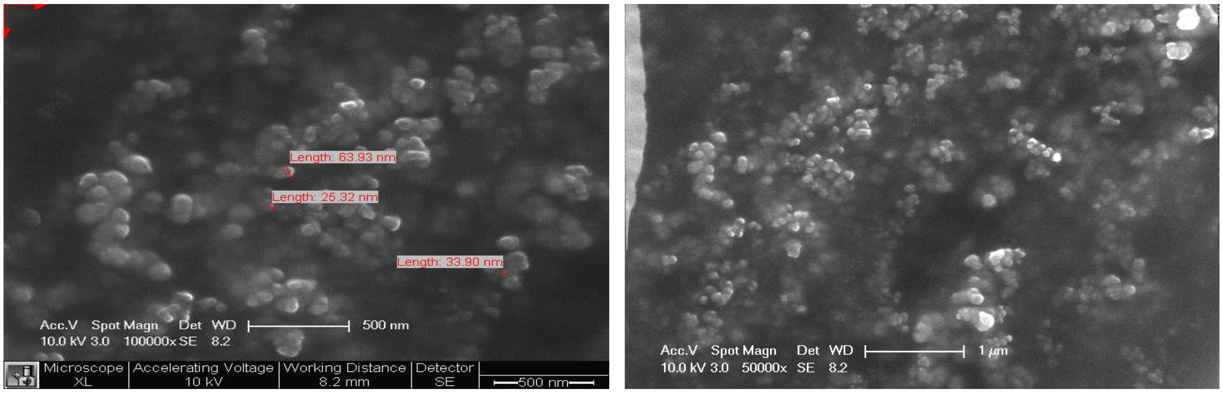

Figure S5. Scanning electron microscopy images of MA-IBU-*co*-PEGMA's nanoparticles prepared by dropwise addition of water to THF solution of the copolymer
